# Insights into Lewy body disease from rare neurometabolic disorders

**DOI:** 10.1007/s00702-021-02355-7

**Published:** 2021-05-30

**Authors:** Daniel Erskine, Johannes Attems

**Affiliations:** 1 Wellcome Centre for Mitochondrial Research, Newcastle, UK; 2 Newcastle University Translational and Clinical Research Institute, Newcastle, UK

**Keywords:** Lewy body, Alpha-synuclein, Iron, Mitochondria, Sphingolipids, Cholesterol

## Abstract

Professor Kurt Jellinger is well known for his seminal work on the neuropathology of age-associated neurodegenerative disorders, particularly Lewy body diseases. However, it is less well known that he also contributed important insights into the neuropathological features of several paediatric neurometabolic diseases, including Alpers–Huttenlocher syndrome, a syndrome of mitochondrial disease caused by *POLG* mutations, and infantile neuroaxonal dystrophy, a phenotype resulting from *PLA2G6* mutations. Despite these rare diseases occurring in early life, they share many important pathological overlaps with age-associated Lewy body disease, particularly dysregulation of α-synuclein. In this review, we describe several neurometabolic diseases linked to Lewy body disease mechanisms, and discuss the wider context to pathological overlaps between neurometabolic and Lewy body diseases. In particular, we will focus on how understanding disease mechanisms in neurometabolic disorders with dysregulated α-synuclein may generate insights into predisposing factors for α-synuclein aggregation in idiopathic Lewy body diseases.

## Introduction

The Lewy body diseases (LBD) include Parkinson’s disease (PD), Parkinson’s disease dementia (PDD), and dementia with Lewy bodies (DLB), all of which lie on a clinical spectrum of motor and cognitive symptoms (Jellinger and Korczyn 2018). PD and PDD present with motor symptoms such as rest tremor, bradykinesia, and unsteady gait that can progress into cognitive impairment, whilst DLB typically present with cognitive features that often develop into parkinsonian features similar to PD (McKeith et al. 2017). The characteristic pathological feature of LBD is intraneuronal inclusions of the protein α-synuclein termed Lewy bodies (McKeith et al. 2017; Spillantini et al. 1997). In addition to the presence of Lewy bodies, LBD cases are also characterised by nigral depigmentation and striatonigral dopaminergic denervation that is thought to underlie the extrapyramidal features that characterise clinical parkinsonism (Jellinger 2012).

The direct pathogenic relevance of Lewy bodies to the neuronal dysfunction and degeneration that characterises LBD is not known (Outeiro et al. 2019). Nevertheless, the observations that mutations in the α-synuclein gene *SNCA* cause familial PD (Polymeropoulos et al. 1997), the presence of α-synuclein in Lewy bodies (Spillantini et al. 1997), and downregulation of α-synuclein levels reducing risk of developing idiopathic PD (Mittal et al. 2017), suggest an important role for α-synuclein in pathological processes in LBD. Despite the putative role of α-synuclein in LBD, many questions remain, including why it aggregates, how (if at all) it induces neuronal dysfunction and degeneration, and thus its suitability as a target for candidate therapeutics.

Neurometabolic diseases typically arise as a result of mutations that induce perturbations in cellular metabolism that prominently affect neurons. Most are very rare and often affect individuals at earlier stages of life than age-associated neurodegenerative disorders; however, they often share overlaps with neuropathological changes observed in individuals with LBD. The present review will discuss metabolic diseases that share neuropathological overlaps with LBD in terms of α-synuclein aggregation, and will focus on PLA2G6-associated neurodegeneration (PLAN), POLG-associated neurodegeneration, Niemann–Pick Type C1, and Krabbe disease. In the present review, we will discuss what is known about shared mechanisms between the neurometabolic diseases of interest and idiopathic Lewy body disease, with a particular focus on whether the proposed pathways may be implicated in α-synuclein dysregulation in idiopathic Lewy body disease.

## PLA2G6-associated neurodegeneration

PLAN is a group of disorders that fall under the umbrella of neurodegeneration with brain iron accumulation and are caused by mutations in the *PLA2G6* gene, the protein product of which is thought to have a role in lipid membrane homeostasis and remodelling (Burke and Dennis 2009). PLAN is typically divided into four sub-types on the basis of clinical features and age of onset: infantile neuroaxonal dystrophy, atypical neuroaxonal dystrophy, adult-onset dystonia-parkinsonism, and autosomal recessive early onset parkinsonism (Guo et al. 2018). Neuroaxonal dystrophy was previously termed “Seitelberger’s disease”, and Prof Kurt Jellinger worked with Prof Franz Seitelberger to study this disease (Jellinger et al. 1968).

Infantile neuroaxonal dystrophy (INAD) presents between 6 months and 3 years of age, with rapidly progressing developmental delay or regression followed by muscle hypotonia and spasticity, leading to a complete loss of voluntary muscle control and death usually by the age of 5–10 years old (Babin et al. 2018). Atypical neuroaxonal dystrophy is characterised by ataxia, rigidity, and spasticity, with later onset from 3 years old to late teens, and slower progression than infantile neuroaxonal dystrophy (Guo et al. 2018). In contrast to infantile neuroaxonal dystrophy, both PLAN sub-types that onset in adulthood, adult-onset dystonia-parkinsonism and autosomal recessive early onset parkinsonism, are characterised by later onset between 20 and 40 years old, a slower rate of clinical deterioration, and responsiveness to dopaminergic agents (Guo et al. 2018). Adult-onset dystonia-parkinsonism is characterised by parkinsonian extrapyramidal features, though there is some heterogeneity in presentation, with neuropsychiatric features such as depression preceding motor symptoms in some cases (Karkheiran et al. 2015).

The characteristic neuropathological features of individuals with *PLA2G6* mutations are prominent neuroaxonal spheroids, iron deposition largely confined to the globus pallidus with variable affectation of the substantia nigra, and widespread neuronal loss (Kruer 2013). Lewy bodies are an invariant finding in all cases reported in the literature with confirmed genetic testing, and were notably present to a severe degree throughout the brain of 8 years old with clinical onset in infancy (Paisan-Ruiz et al. 2012). Lewy body diseases are normally associated with advancing age, and incidental Lewy bodies are typically only observed in individuals over the age of 60 (Frigerio et al. 2011; Outeiro et al. 2019); therefore, the presence and severity of Lewy body pathology in individuals as young as 8 years old suggests an association between Lewy body pathology and *PLA2G6* mutations. As a result, and given that Lewy body formation in PLAN appear to follow Braak’s pathological staging scheme for Lewy body pathology in PD (Braak et al. 2003; Paisan-Ruiz et al. 2012), it has been suggested that INAD should be considered to lie on the α-synucleinopathy spectrum (Jellinger 2003). It is also notable that many cases of PLAN also manifested tau pathology, though this was a less invariant finding than Lewy body pathology as the 8 years old with severe Lewy body pathology did not manifest concomitant tau pathology (Paisan-Ruiz et al. 2012). Nevertheless, given the putative potentiating effect of α-synuclein on tau pathology (Bassil et al. 2021), and tau on α-synuclein pathology (Dasari et al. 2019), these interactions may contribute to pathological and clinical features of PLAN.

How *PLA2G6* mutations induce the aggregation of α-synuclein is not clear, but as infantile neuroaxonal dystrophy is primarily characterised as an iron storage disorder, and iron dyshomeostasis has been implicated in α-synuclein aggregation (Xiao et al. 2018), one could speculate that elevated iron levels could be associated with α-synuclein aggregation in infantile neuroaxonal dystrophy. However, knockdown of *PLA2G6* in drosophila led to changes in the composition of the phospholipid bilayer of neuronal cell membranes towards lipids with shorter acyl chains and increased membrane curvature, leading to the dissociation of α-synuclein from cell membranes and increased fibrillisation (Mori et al. 2019). Consistent with this proposition, *PLA2G6* knockdown in mice led to α-synuclein aggregation in mitochondria (Sumi-Akamaru et al. 2016), perhaps due to altered membrane composition and subsequent dissociation of α-synuclein from mitochondrial membranes (Shen et al. 2014). Therefore, one could speculate that *PLA2G6* mutations affect the membrane-binding propensity of α-synuclein, causing its dissociation and subsequent loss of alpha-helical structure, and aggregation in a concentration-dependent manner (Outeiro et al. 2019).

## POLG-associated neurodegeneration

**Fig. 1:**
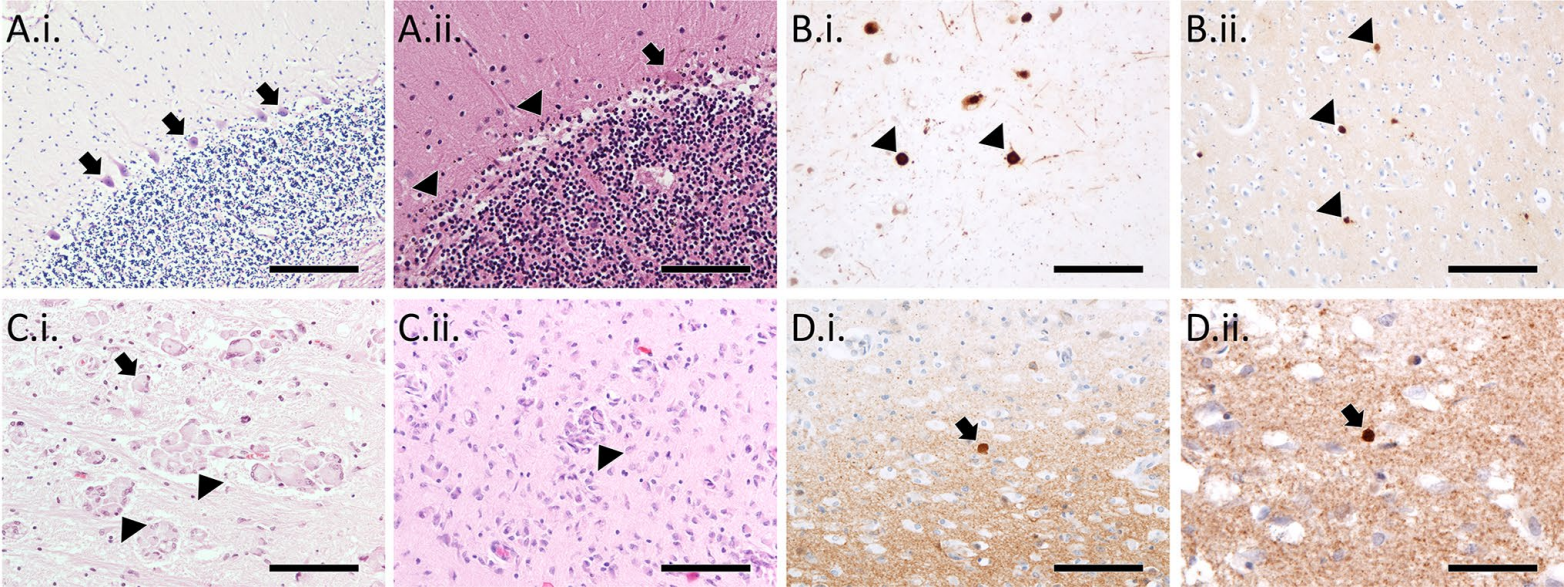
In contrast to control cerebellum, which shows a clear layer of Purkinje cells (arrows) on H&E stain (**A.i.**), individuals with *POLG* mutations typically show a loss of Purkinje cells, with some dystrophic remnants of Purkinje cells (arrow), alongside associated Bergmann gliosis (arrowheads; **A.ii.**). The substantia nigra of a 59 year old male with a *POLG* mutation shows α-synuclein-immunoreactive Lewy bodies (arrowheads; **B.i.**), as does the temporal cortex of a 79 year old male with a *POLG* mutation (arrowheads; **B.ii.**). Krabbe disease patients show clusters of lipid-filled multi-nucleated globoid cells (arrowheads) and single mononucleated foamy macrophages (arrows) in the medial lemniscus of a 10 month old male (**C.i.**) and occipital white matter of a 9 month old male (**C.ii.**). α-Synuclein immunohistochemistry of Krabbe disease cases demonstrates spherical inclusions in frontal cortex of a 10 month old male (**D.i.**) and the putamen of a 12 month old female (**D.ii.**). Scale bars = 200 µm (**A.i.**, **B.i.**, **B.ii.**), 100 µm (**A.ii.**, **C.i.**, **C.ii.**, **D.i.**), 50 µm (**D.ii.**)

Mutations in *POLG* give rise to several clinical syndromes, including: Alpers–Huttenlocher syndrome (AHS), myocerebrohepatopathy spectrum (MCHS), myoclonic epilepsy myopathy sensory ataxia (MEMSA), ataxia neuropathy spectrum (ANS), and progressive external ophthalmoplegia (PEO) (Stumpf et al. 2013). Typically, AHS and MCHS onset in infancy or childhood, both with liver dysfunction and developmental delay, though AHS is also characterised by epilepsy, particularly in the occipital region, progressing to *epilepsia partialis continua* and *status epilepticus* (Wolf et al. 2009). MEMSA onsets in adolescence or adulthood and is characterised by epilepsy, myopathy and ataxia (Van Goethem et al. 2003). ANS onsets in early adolescence or adulthood and is characterised by ataxia and neuropathy, but without myopathy, and some may also have PEO (Van Goethem et al. 2004). Finally, PEO onsets in adulthood with progressive weakening of extraocular eye muscles leading to ptosis and reduced eye movement, though myopathy is often observed in these individuals (Van Goethem et al. 2001). As with many rare diseases, neuropathological data on individuals harbouring *POLG* mutations are limited by the relatively few reports in the literature. However, individuals with *POLG* mutations have varied pathological features, ranging from severe necrosis of the occipital cortex, particularly in AHS, to cortical laminar necrosis, with loss of cerebellar Purkinje cells an almost invariant finding across phenotypes (Fig. 1A.i., A.ii.).

*POLG* mutations have been linked to degeneration of the nigrostriatal system similar to PD with concomitant, though variable, parkinsonism. One study that conducted neuropathological examinations of four *POLG* mutation carriers identified severe deficiency of Complex I of the mitochondrial respiratory chain in substantia nigra neurons, but that this was not associated with clinical parkinsonism, whereas loss of nigral neurons was a good correlate of parkinsonian severity (Palin et al. 2013). In contrast, another study in six younger *POLG* cases confirmed severe Complex I deficiency and marked nigral neuronal loss but an absence of parkinsonian clinical features (Tzoulis et al. 2013). Although initial reports of parkinsonism in PEO cases indicated an absence of Lewy bodies in two cases, we have subsequently reported a higher prevalence of Lewy body pathology in a prospective series of older mitochondrial disease cases (Fig. 1B.i., B.ii.), particularly those with nuclear DNA mutations such as *POLG*, than in a comparable and older control population (Erskine et al. 2020). This study did not show consistently higher levels of Alzheimer-type pathology compared to that observed in normal ageing, and Lewy body pathology appeared to conform to Braak’s scheme for the distribution of Lewy bodies in PD (Braak et al. 2003). Consistent with the proposition that POLG mutations may increase vulnerability to Lewy body pathology, stem cells derived from a patient with a *POLG* mutation had increased levels of high molecular weight α-synuclein and higher levels of α-synuclein phosphorylated at serine 129 (Chumarina et al. 2019).

There are many potential mechanisms that could explain why individuals with *POLG* mutations would have increased risk of developing Lewy body pathology, including the long-established relationship between Complex I inhibition and α-synuclein aggregation (Cannon et al. 2009), excessive production of reactive oxygen species leading to the accumulation of degradation-resistant oxidised α-synuclein (Martinez-Vicente et al. 2008), and increasingly oxidised intracellular environments leading to increased unbound α-synuclein through reductions in its binding partners (Scarlata and Golebiewska 2014). The mitochondrial lipid cardiolipin has been demonstrated to stabilise monomeric α-synuclein on mitochondrial inner membranes (Ryan et al. 2018), but is highly vulnerable to peroxidation secondary to excessive production of reactive oxygen species (Paradies et al. 2014) that reportedly occurs in neuronal cells generated from *POLG* mutation carriers (Liang et al. 2020). Therefore, it is also plausible to suggest that altered mitochondrial membrane dynamics could lead to increased levels of unbound α-synuclein within neurons, somewhat similar to that proposed for *PLA2G6* mutations.

## Niemann–Pick type C1

Niemann–Pick disease is an umbrella term for a group of autosomal recessive lysosomal disorders characterised by the accumulation of undegraded lipids within affected cells (Santos-Lozano et al. 2015). Niemann–Pick type A and B differ from C and D on the basis of the affected mechanisms, with A and B resulting from sphingomyelinase deficiency and C and resulting from mutations in proteins involved in intracellular lipid and cholesterol trafficking (Sun, 2018). Niemann–Pick C1 results from mutations in *NPC1*, a lysosomal membrane protein, the loss of which induces defects in intracellular lipid trafficking and the accumulation of unesterified cholesterol and other sphingolipid species (Yu et al. 2014). Niemann–Pick C1 can present across childhood and adolescence, with early onset associated with more rapid progression, and typically involving hepatosplenomegaly, developmental delay, ataxia, hypotonia, and saccadic abnormalities (Sun 2018).

Neuropathologically, Niemann–Pick C1 is characterised by widespread accumulation of gangliosides and unesterified cholesterol in neurons, alongside meganeurites often larger than the soma from which they emanate (Zervas et al. 2001). There have been several reports of α-synuclein accumulation in Niemann–Pick C1 cases, from one case study reporting Lewy bodies (Chiba et al. 2014), to a case series of 12 individuals, of which nine had intraneuronal α-synuclein aggregation characterised as “pre-Lewy bodies”, whilst in two cases aged 23 and 32, it was typical Lewy body pathology (Saito et al. 2004). Therefore, although Niemann–Pick C1 appears associated with the accumulation of α-synuclein in most cases, it is not clear how this related to Lewy body formation, nor whether they conform to the distribution of Lewy body pathology in PD (Braak et al. 2003). However, given the young age of cases manifesting Lewy bodies, much younger than the age at which incidental Lewy body disease typically occurs, one could suggest an association between *NPC1* mutations and α-synuclein aggregation. It is also notable that tau pathology was also observed in 10/12 Niemann–Pick cases with α-synuclein pathology noted previously, where its abundance appeared to closely mirror that of α-synuclein, and with which it was frequently co-localised (Saito et al. 2004).

There are several potential mechanisms that could underlie the increased levels of aggregated α-synuclein in Niemann–Pick Type C1. In addition to cholesterol, lipidomic analyses have demonstrated reductions of galactosylceramide alongside striking increases in cholesterol, glucosylceramide, lactosylceramide, gangliotriaosylceramide, and GM2 and GM3 gangliosides in Niemann–Pick type C1 (Vanier 1999). Glucosylceramide has been widely studied in the context of Lewy body diseases, where it has been demonstrated to precipitate the toxic conversion of α-synuclein (Zunke et al. 2018), and thus, its elevation in Niemann–Pick type C1 may contribute vulnerability to α-synuclein aggregation. GM2 gangliosides have also been associated with α-synuclein pathology in mouse models (Suzuki et al. 2003, 2007) and GM3 gangliosides have been demonstrated to induce α-synuclein fibrillisation in vitro (Gaspar et al. 2018). Therefore, there are several plausible links between lipids accumulated in Niemann–Pick type C1 and the aggregation of α-synuclein.

## Krabbe disease

Krabbe disease is a rare autosomal recessive neurodegenerative disorder resulting from mutations in the *GALC* gene, which encodes the lipid-degrading lysosomal enzyme β-galactocerebrosidase (Graziano and Cardile 2015). 95% of cases have onset within the first 6 months of life, presenting with hyperirritability, hypersensitivity to external stimuli, and stiffness of limbs. Infantile cases progress quickly with marked psychomotor regression and hypotonicity, leading to a state of decerebrate posture and complete non-responsiveness, prior to death typically before the age of 2 years old (Hagberg et al. 1963). Later onset cases have been described, though their onset and progression is more variable, ranging from late infantile through to juvenile and even adult onset (Debs et al. 2013; Lyon et al. 1991). The classic neuropathological feature of Krabbe disease is spongiosis of the white matter and the presence of swollen lipid-laden macrophages termed globoid cells (Fig. 1C.i., C.ii.) (Itoh et al. 2002). The characteristic leukodystrophy of Krabbe disease is thought to result from the accumulation of the toxic lipid psychosine, a precursor of galactosylceramide, a substrate of the enzyme encoded by *GALC* (Spassieva and Bieberich 2016).

Only three infantile Krabbe disease cases have been evaluated for α-synuclein pathology in the literature, with all demonstrating Thioflavin-S and α-synuclein-positive spherical inclusions reminiscent of Lewy bodies in frontal cortical tissue (Fig. 1D.i., D.ii.) (Smith et al. 2014). These findings are notable both due to the very young age of the Krabbe disease patients in which α-synuclein pathology was observed, but also as variants in *GALC* are associated with increased risk of developing idiopathic Parkinson’s disease, this indicates a direct mechanistic link between Krabbe disease and age-associated Lewy body diseases (Kia et al. 2021; Li et al. 2018). Given that α-synuclein pathology has only been evaluated in one brain region in human Krabbe disease brains, it is not possible to determine the extent to which it overlaps with the distribution of Lewy body pathology in PD. Furthermore, no study to our knowledge has evaluated whether concomitant Alzheimer-type pathology is present in Krabbe disease brains.

There are several plausible mechanisms that may account for α-synuclein pathology in Krabbe disease; first, psychosine has been demonstrated to interact with, and induce the fibrillisation of, α-synuclein in vitro (Abdelkarim et al. 2018). However, psychosine also induces cholesterol mislocalisation and alterations to membrane curvature (D'Auria et al. 2017; Hawkins-Salsbury et al. 2013), which may promote α-synuclein membrane dissociation and subsequent accumulation.

## Implications for idiopathic Lewy body disease

Rare neurometabolic diseases with α-synuclein pathology offer a potentially unique insight into the formation of Lewy bodies in idiopathic Lewy body disease, given that their aetiology is well known and thus mechanisms that may give rise to α-synuclein aggregation can be inferred. If processes affected in the described neurometabolic disease are also known to be perturbed in idiopathic Lewy body disease, then one could speculate that they may contribute to the formation of Lewy bodies and may be worthy of further study to better understand the pathogenesis of Lewy body disease.

There are conflicting reports about the role of variants in *PLA2G6* and risk of PD, with some demonstrating an association (Liu et al. 2020a, b) and others not (Liu et al. 2020a, b). However, a study using cells obtained from idiopathic PD patients demonstrated deficient activity of iPLA2, the protein product of *PLA2G6* (Zhou et al. 2016), and it has been reported to be located within Lewy bodies of idiopathic PD cases (Miki et al. 2017). Therefore, although limited, there is some evidence for alterations to *PLA2G6*-associated function in idiopathic LBD; however, given that Lewy bodies are an invariant finding in all PLAN cases in the literature, further understanding of how iPLA2 is altered in idiopathic Lewy body disease is a pressing issue.

Similar to *PLA2G6*, *POLG* variants are not thought to be associated with increased risk of developing idiopathic Lewy body disease (Bentley et al. 2014; Hudson et al. 2009; Tiangyou et al. 2006). However, *POLG* is a mitochondrial DNA polymerase, the dysfunction of which has a profound impact on the mitochondrial respiratory chain (Lax et al. 2016), and alterations to the mitochondrial respiratory chain have been consistently observed in PD and DLB (Hatton et al. 2020; Reeve et al. 2012; Schapira et al. 1990), which may point to some shared aetiological factors. In contrast to the mitochondrial respiratory chain, no study to our knowledge has investigated whether alterations to cardiolipin occur in Lewy body disease despite increasing interest in this area (Gilmozzi et al. 2020). Therefore, it remains unclear whether  cellular changes resulting from *POLG* mutations that are putatively related to α-synuclein aggregation are also observed in Lewy body disease.

Natural variants in *NPC1* are not associated with increased risk of PD (Ouled Amar Bencheikh et al. 2020; Zech et al. 2013); however, some of the substrates that accumulate in Niemann–Pick Type C1, such as cholesterol, have been identified as dysregulated in idiopathic PD (Huang et al. 2019; Park et al. 2021) and DLB (Bettcher et al. 2017; Bosco et al. 2006). As with Niemann–Pick type C1, there is evidence for alterations to GM3 gangliosides in Lewy body diseases, with studies demonstrating elevations in plasma (Chan et al. 2017) and cerebrospinal fluid (Huebecker et al. 2019) in PD. There is also evidence that upregulation of β-hexosaminidase, an enzyme responsible for degradation of GM2 gangliosides, protects dopaminergic neurons from α-synuclein toxicity, implying a role for GM2 gangliosides in α-synuclein-mediated neurodegeneration (Brekk et al. 2020). Furthermore, glucosylceramide, which is elevated in Niemann–Pick type C1, has been demonstrated to be elevated in PD (Mielke et al. 2013), where it has been demonstrated to induce α-synuclein aggregation (Zunke et al. 2018) and enhance neuronal susceptibility to α-synuclein toxicity (Henderson et al. 2020). Taken together, these findings suggest that whilst *NPC1* is not a risk factor for idiopathic Lewy body diseases, there is evidence to suggest dysregulation of lipid species associated with Niemann–Pick Type C1.

Unlike *PLA2G6*, *POLG*, and *NPC1*, multiple large studies have consistently reported that *GALC* variants are associated with PD risk (Kia et al. 2021; Li et al. 2018). Furthermore, there is direct evidence that psychosine, the lipid that accumulates in Krabbe disease, directly interacts with α-synuclein and induces its fibrillisation in vitro (Abdelkarim et al. 2018; Smith et al. 2014), and is elevated in PD brain tissue lysates (Marshall et al. 2018). Taken together, these findings highlight plausible and direct mechanisms through which dysregulation of psychosine may contribute to α-synuclein pathology in Krabbe disease and idiopathic Lewy body disease.

## Discussion

The discussed neurometabolic diseases typically have a relatively young onset, in contrast to the age-associated presentation of idiopathic Lewy body diseases. Nevertheless, despite incidental Lewy bodies typically occurring over the age of 60 years (Gilmozzi et al. 2020), it is notable that many of the discussed metabolic diseases manifest α-synuclein aggregates at a much younger age. Given that Lewy body pathology occurs at a much younger age in individuals with particular neurometabolic diseases than occurs incidentally, one could quite reasonably suggest that α-synuclein aggregation is related to the underlying metabolic dysfunction that gives rise to the primary pathology. Therefore, understanding the processes that may contribute to α-synuclein aggregation in neurometabolic diseases, where causal disease mechanisms are already well characterised, may provide unique insights into pathological processes that contribute to Lewy body formation in idiopathic Lewy body disease.

The present discussion has highlighted four rare neurometabolic disorders with α-synuclein accumulation, all of which have alterations to particular lipid species that are also reportedly dysregulated in idiopathic Lewy body disease. Therefore, it is tempting to speculate that alterations to lipid metabolism may contribute to α-synuclein aggregation, either by direct lipid–protein interactions, as with psychosine or GM3 gangliosides, or indirectly by reducing membrane binding and thus increasing the abundance of free α-synuclein within neurons, as with *PLA2G6* and cardiolipin. These are not trivial matters of enquiry as many candidate disease-modifying therapies in development for idiopathic Lewy body disease target the aggregation of α-synuclein. However, if α-synuclein aggregation in idiopathic Lewy body disease results from an underlying metabolic deficit, as it appears to do in some rare neurometabolic disorders, then such efforts are unlikely to be effective as they would not address the underlying metabolic dysfunction that led to α-synuclein aggregation, and which likely contributes to neural dysfunction and degeneration.

Although there is a renewed interest in the role of lipids in Lewy body diseases (Fanning et al. 2020), there is a pressing need to better understand changes to lipid species in post-mortem Lewy body disease brain tissue and how these may contribute to Lewy body formation. One potential barrier to this is that lipids are more difficult to study in brain tissue than proteins, and the methods involved often require tissue homogenates meaning that topographical information is lost compared to histological preparations. There is also a pressing need to better understand the extent to which α-synuclein aggregates in neurometabolic diseases recapitulate key pathogenic features of Lewy body diseases, such as increased levels of phosphorylation at serine 129 and the ability to seed aggregation of monomeric α-synuclein. Answering these questions may help gain further insights into α-synucleinopathies and determine the extent to which these diseases at the opposite ends of life share key pathogenic mechanisms.
